# Supplementary material for: Supervised Learning With First-to-Spike Decoding in Multilayer Spiking Neural Networks
Source: Front Comput Neurosci. 2021 Apr 12;15:617862. doi: 10.3389/fncom.2021.617862 (PMC8072060; doi:10.3389/fncom.2021.617862)
Supplement: Supplementary file 1 [file Data_Sheet_1.PDF]

Supplementary Material

1 EXTENDING THE LEARNING RULE

The procedure used to derive weight updates in multilayer SNNs is also extensible to deeper network architectures. For demonstrative purposes, we consider a multilayer SNN containing two hidden layers, and derive weight updates for hidden neurons residing in the third-last layer, i.e. for layer $l = L - 2$; refer to Fig S1 for a schematic of this network structure, and for the notation we use from this point onwards.

Gradient descent is used to minimise the network's cross-entropy loss (given by Eq (7) in the main text) with respect to weights projecting onto layer $L - 2$, according to

$$\Delta w_{ij}^{L-2} = -\eta \frac{\partial C(\mathbf{y}, \mathbf{a}^L)}{\partial w_{ij}^{L-2}} \,. \tag{S1}$$

Using the chain rule, the gradient term is expanded as follows:

$$\begin{aligned} \frac{\partial C(\mathbf{y}, \mathbf{a}^L)}{\partial w_{ij}^{L-2}} &= \sum_{i' \in I'} \frac{\partial C(\mathbf{y}, \mathbf{a}^L)}{\partial u_{i'}^L} \frac{\partial u_{i'}^L(t)}{\partial w_{ij}^{L-2}} \bigg|_{t=\tau_{i'}} \\ &= \sum_{i' \in I'} \delta_{i'}^L \frac{\partial u_{i'}^L(t)}{\partial w_{ij}^{L-2}} \bigg|_{t=\tau_{i'}} \,, \end{aligned} \tag{S2}$$

where for convenience we use the identity of the output error signal, $\delta_{i'}^L$, defined by Eq (14) in the main text. Hence, using the previously defined SRM$_0$ (Eq (4), main text), the gradient of the i'^{th} output neuron's

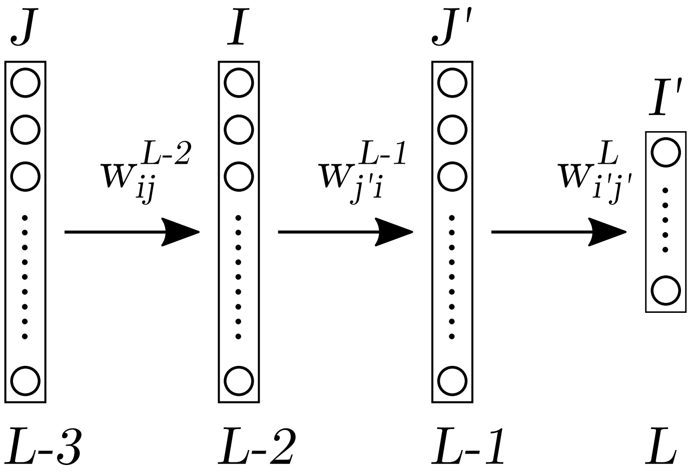

Figure S1. Schematic of the multilayer SNN architecture considered here, containing two hidden layers. The network is feedforward with all-to-all connectivity between adjacent layers. From the last layer, L, to the first layer, $L - 3$, neurons are respectively indexed: $i' \in I'$, $j' \in J'$, $i \in I$, and $j \in J$. Synaptic weights are denoted with respect to the postsynaptic layer, for example w_{ij}^{L-2} is the weight projecting from presynaptic neuron j onto postsynaptic neuron i residing in layer $L - 2$.

membrane potential is given by

$$\left.\frac{\partial u_{i'}^{L}(t)}{\partial w_{ij}^{L-2}}\right|_{t=\tau_{i'}} = \sum_{j'\in\Gamma_{i'}^{L}} w_{i'j'}^{L}\frac{\partial}{\partial w_{ij}^{L-2}}\left(\epsilon * S_{j'}^{L-1}\right)(\tau_{i'})$$
$$= \sum_{j'\in\Gamma_{i'}^{L}} w_{i'j'}^{L}\left(\epsilon * \frac{\partial S_{j'}^{L-1}}{\partial w_{ij}^{L-2}}\right)(\tau_{i'}) \,, \tag{S3}$$

where $\Gamma_{i'}^{L}$ is the set of immediate neural predecessors of a last layer neuron i'. As in section 2.2.5 of the main text, we substitute the ill-defined gradient of a hidden layer spike train with the gradient of its expected value, and consider spikes to be distributed according to an underlying, instantaneous firing rate: $\rho_{j'}^{L-1}(t) = g(u_{j'}^{L-1}(t))$. Hence, following this previously described procedure, we arrive at the following expression for the gradient of the expected hidden spike train:

$$\frac{\partial\left\langle S_{j'}^{L-1}(t)\right\rangle_{S_{j'}^{L-1}|L-2,j'}}{\partial w_{ij}^{L-2}} = \delta_D(t-\hat{t})\frac{\partial\rho_{j'}^{L-1}(t|L-2,j')}{\partial w_{ij}^{L-2}} \,, \tag{S4}$$

where the expected spike train is conditioned on activity in the previous layer, $L-2$, and its own last firing time, $\hat{t}_{j'}$. As before, δ_D is the Dirac-delta function: depending on an arbitrary, last firing time, $\hat{t}$. Combining the above with Eqs (4) and (23) in the main text, we find

$$\frac{\partial\left\langle S_{j'}^{L-1}(t)\right\rangle_{S_{j'}^{L-1}|L-2,j'}}{\partial w_{ij}^{L-2}} = \frac{1}{\Delta u}\delta_D(t-\hat{t})\rho_{j'}^{L-1}(t|L-2,j')\frac{\partial u_{j'}^{L-1}(t)}{\partial w_{ij}^{L-2}}$$
$$= \frac{1}{\Delta u}\left\langle S_{j'}^{L-1}(t)\frac{\partial u_{j'}^{L-1}(t)}{\partial w_{ij}^{L-2}}\right\rangle_{S_{j'}^{L-1}|L-2,j'} . \tag{S5}$$

The gradient of the j'^{th} neuron's membrane potential is expanded with respect to the previous layer as follows:

$$\frac{\partial u_{j'}^{L-1}(t)}{\partial w_{ij}^{L-2}} = w_{j'i}^{L-1}\frac{\partial}{\partial w_{ij}^{L-2}}\left(\epsilon * S_{i}^{L-2}\right)(t)$$
$$= w_{j'i}^{L-1}\left(\epsilon * \frac{\partial S_{i}^{L-2}}{\partial w_{ij}^{L-2}}\right)(t) \,, \tag{S6}$$

where all spike train gradients in layer $L-2$ except for i^{th} one do not depend on w_{ij}^{L-2}, and therefore vanish. As usual, the gradient of a hidden spike train is substituted with the gradient of its expected value,

conditioned on the neuron's last firing time and its received spike trains from the previous layer:

$$\frac{\partial \left\langle S_i^{L-2}(t) \right\rangle_{S_i^{L-2}|L-3,i}}{\partial w_{ij}^{L-2}} = \delta_D(t-\hat{t}) \frac{\partial \rho_i^{L-2}(t|L-3,i)}{\partial w_{ij}^{L-2}}$$
$$= \frac{1}{\Delta u} \left\langle S_i^{L-2}(t) \frac{\partial u_i^{L-2}(t)}{\partial w_{ij}^{L-2}} \right\rangle_{S_i^{L-2}|L-3,i} , \tag{S7}$$

where hidden spikes in layer $L-2$ are distributed according to ρ_i^{L-2}. The gradient of the neuron's membrane potential is then determined as follows:

$$\frac{\partial u_i^{L-2}(t)}{\partial w_{ij}^{L-2}} = \left(\epsilon * S_j^{L-3} \right)(t) . \tag{S8}$$

Hence, combining Eqs (S5) to (S8) provides the gradient of the expected value of the hidden spike train in layer $L-1$:

$$\frac{\partial \left\langle S_{j'}^{L-1}(t) \right\rangle_{S_{j'}^{L-1}|L-2,j'}}{\partial w_{ij}^{L-2}}$$
$$= \frac{w_{j'i}^{L-1}}{(\Delta u)^2} \left\langle S_{j'}^{L-1}(t) \left(\epsilon * \left\langle S_i^{L-2} \left(\epsilon * S_j^{L-3} \right) \right\rangle_{S_i^{L-2}|L-3,i} \right)(t) \right\rangle_{S_{j'}^{L-1}|L-2,j'} . \tag{S9}$$

As usual, we estimate the above expected gradient by sampling hidden spike trains on each simulation run. Therefore, dropping the expectation operators provides the gradient estimator:

$$\frac{\partial \left\langle S_{j'}^{L-1}(t) \right\rangle_{S_{j'}^{L-1}|L-2,j'}}{\partial w_{ij}^{L-2}} \approx \frac{w_{j'i}^{L-1}}{(\Delta u)^2} S_{j'}^{L-1}(t) \left(\epsilon * \left[S_i^{L-2} \left(\epsilon * S_j^{L-3} \right) \right] \right)(t) . \tag{S10}$$

Substituting the above into Eq (S3) yields the gradient of a neuron's membrane potential in the final layer:

$$\left. \frac{\partial u_{i'}^L(t)}{\partial w_{ij}^{L-2}} \right|_{t=\tau_{i'}} = \frac{1}{(\Delta u)^2} \sum_{j' \in \Gamma_{i'}^L} w_{i'j'}^L w_{j'i}^{L-1} \left(\epsilon * \left[S_{j'}^{L-1} \left(\epsilon * \left[S_i^{L-2} \left(\epsilon * S_j^{L-3} \right) \right] \right) \right] \right)(\tau_{i'}) , \tag{S11}$$

Combining the above with Eqs (S1) and (S2) gives rise to the weight update rule for the third-last layer:

$$\Delta w_{ij}^{L-2} = -\frac{\eta}{(\Delta u)^2} \sum_{i' \in I'} \delta_{i'}^L \sum_{j' \in \Gamma_{i'}^L} w_{i'j'}^L w_{j'i}^{L-1} \left(\epsilon * \left[S_{j'}^{L-1} \left(\epsilon * \left[S_i^{L-2} \left(\epsilon * S_j^{L-3} \right) \right] \right) \right] \right)(\tau_{i'}) . \tag{S12}$$

The above weight update formula depends on chained convolution operations, deriving from the intermediate synaptic processes occuring between distant, upstream spiking events and downstream target activity. By following a similar approach to the above steps, hidden weight updates for deeper network architectures can also be derived sharing this pattern.
